# CyanoTag: Discovery of protein function facilitated by high-throughput endogenous tagging in a photosynthetic prokaryote

**DOI:** 10.1126/sciadv.adp6599

**Published:** 2025-02-07

**Authors:** Abigail J. Perrin, Matthew Dowson, Katharine Davis, Onyou Nam, Adam A. Dowle, Grant Calder, Victoria J. Springthorpe, Guoyan Zhao, Luke C. M. Mackinder

**Affiliations:** ^1^ Department of Biology, University of York, York YO10 5DD, UK.; ^2^ Centre for Novel Agricultural Products (CNAP), Department of Biology, University of York, York YO10 5DD, UK.

## Abstract

Despite their importance to aquatic ecosystems, global carbon cycling, and sustainable bioindustries, the genomes of photosynthetic bacteria contain large numbers of uncharacterized genes. Here, we develop high-throughput endogenous fluorescent protein tagging in the cyanobacterium *Synechococcus elongatus* PCC 7942. From 400 targets, we successfully tag over 330 proteins corresponding to >10% of the proteome. We use this collection to determine subcellular localization, relative protein abundances, and protein-protein interaction networks, providing biological insights into diverse processes—from photosynthesis to cell division. We build a high-confidence protein-protein interaction map for the major components of photosynthesis, associating previously uncharacterized proteins with different complexes and processes. In response to light changes, we visualize, on second timescales, the reversible formation, growth, and fusion of puncta by two Calvin cycle proteins, suggesting that biomolecular condensation provides spatiotemporal control of the Calvin cycle in cyanobacteria. We envision that these insights, cell lines, and optimized methods will facilitate rapid advances in cyanobacteria biology and, more broadly, all photosynthetic life.

## Teaser

A resource for protein functional discovery in cyanobacteria advances understanding of photosynthesis and beyond.

## INTRODUCTION

Cyanobacteria fulfill essential roles in maintaining Earth’s atmosphere and ecosystems. These photosynthetic bacteria make a substantial contribution to global CO_2_ fixation, O_2_ production, and nutrient cycling (*1*, *2*). They form intricate connections and symbioses across diverse ecosystems with their imbalance leading to disastrous effects (*3*). Through ancient primary endosymbiosis events that led to the development of chloroplasts in eukaryotes, cyanobacteria have shaped the evolution of hugely diverse plant and algal species (*4*). Many photosynthetic genes and central metabolic processes remain conserved between cyanobacteria and eukaryotic chloroplasts. Thus, understanding cyanobacterial cell biology can contribute to advances in knowledge of fundamental cellular processes, chloroplast biology, and endeavors in photosynthetic engineering of plants (*5*, *6*). In addition, their diversity, genetic tractability, and photosynthetic capability position cyanobacteria as promising chassis for green biotechnology applications, including carbon capture and high-value product manufacture (*7*).

*Synechococcus elongatus* PCC 7942 (*S. elongatus* hereafter) is a freshwater cyanobacterium, the first to be transformed with exogenous DNA (*8*) and now a commonly used model organism. Its compact (~2.7 mega–base pairs) genome and elongated morphology with identifiable cellular structures also make it an attractive organism for study. Despite the global importance of cyanobacteria and their widespread use in research, a notably small proportion of cyanobacterial proteins have been characterized; over a quarter of *S. elongatus* genes lack any functional annotation, with most of the remainder annotated only via bioinformatic methods (through genetic homology to characterized genes in other species). Hence, there are large gaps remaining in our understanding of fundamental biology relating to photosynthesis, cell division, central metabolism, and more.

The use of large-scale tagging approaches has led to substantial insights into model systems (*9*). Most studies to date have focused on model organisms including yeast (*10*, *11*), *Caenorhabditis elegans* (*12*), Drosophila (*13*), and human cells (*14*). Some more recent studies have started to develop and apply large-scale localization approaches to other eukaryotic groups including the parasite *Trypanosoma brucei* (*15*) and the photosynthetic alga *Chlamydomonas reinhardtii* (*16*, *17*). Outside of *Escherichia coli* (*18*), large-scale tagging has had limited application in bacterial systems and has yet to be applied to a photosynthetic bacteria. Furthermore, the application of endogenous, scarless fluorescent protein tagging via chromosomal integration and subsequent removal of markers has yet to be applied at scale to photosynthetic systems. To enable the rapid advancement of our knowledge of photosynthesis and cyanobacterial cell biology, we built a pipeline for the generation of a library of *S. elongatus* lines expressing fluorescently tagged proteins amenable for use in protein localization, expression, and interaction studies. Our approach involves the scarless tagging of each gene at its native locus, maintaining endogenous regulatory regions and allowing tag fluorescence to be used as a proxy for protein abundance.

Here, we report the tagging and further characterization of 330 protein-coding genes (around 12% of the *S. elongatus* genome) and examples of biological insights this has already provided. We also present a preliminary protein interactome based on affinity purification experiments where we detected over half the 2714 known proteins in this organism. The interactome map is the first of its kind for a cyanobacterial species and includes 369 high-confidence protein-protein interactions. As we expand our libraries toward coverage of the entire *S. elongatus* proteome, we are sharing the growing datasets via an interactive web tool (https://morf-db.org/projects/York-Mackinder-Lab/MORF000032). We anticipate our cell libraries, associated data, and optimized methods (see the Supplementary Materials) being valuable resources for research communities, providing insights into both known processes and uncharacterized proteins in cyanobacteria and beyond.

## RESULTS

### Development of CyanoTag, a high-throughput protein tagging platform

**Fig. 1.**
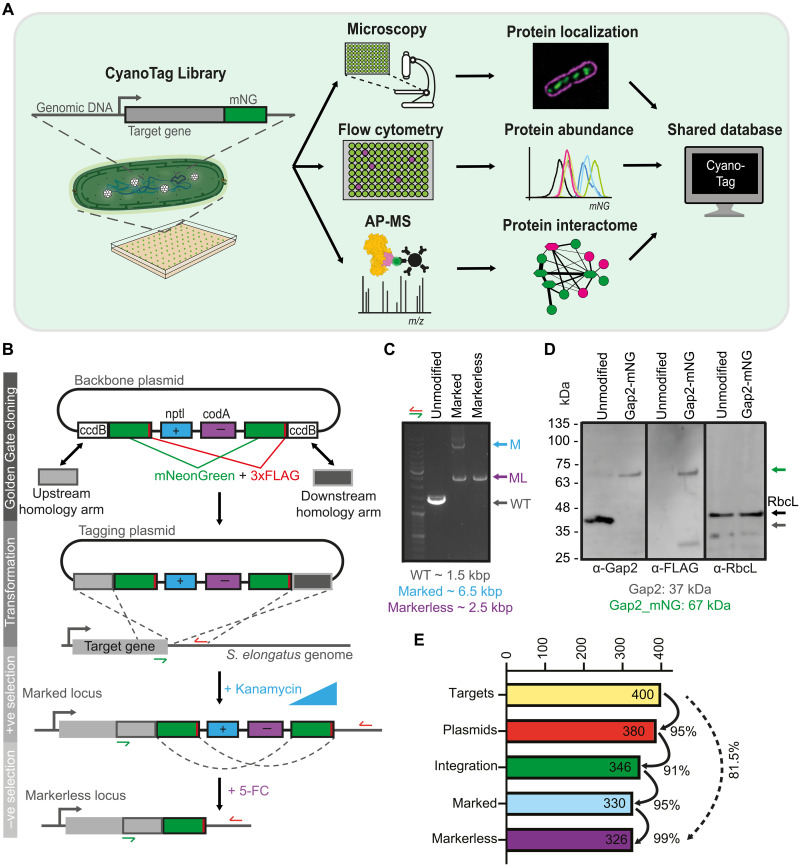
High-throughput fluorescent protein tagging pipeline in *S. elongatus* PCC 7942. (**A**) Overview of the multipurpose 96-well pipeline, which can be used to tag proteins of interest at their native loci for the study of protein distribution, protein abundance, and protein-protein interactions. AP-MS, affinity purification–mass spectrometry. (**B**) Schematic showing the pipeline for high-throughput protein tagging. Two homology arms (HAs) are amplified by PCR from genomic DNA, and a BsaI- or BspQI-dependent Golden Gate cloning reaction is used to insert these HAs in place of two cytotoxin-encoding (*ccdB*) genes in a backbone plasmid, which contains a series of tagging and selection modules. These are an mNG fluorescent protein and 3xFLAG epitope (mNG/3xFLAG) tagging module, a kanamycin antibiotic selection marker (*nptI*), and a negative selection marker (*codA*) followed by a second copy of the mNG/3xFLAG module. Following transformation of *S. elongatus* PCC 7942 with these plasmids and selection with kanamycin, homologous recombination leads to integration of the tagging and selection modules at the 3′ end of the target gene and the generation of marked mutants. Markerless mutants are generated via a second recombination event, enhanced by selection with 5-FC, between the two mNG/3xFLAG modules. (**C**) Example of colony PCRs showing the changes in locus size as homoplasmic marked and markerless mutants are generated in the CyanoTag pipeline. We observe a degree of “spontaneous” marker removal before the addition of 5-FC in most marked lines. Primer locations are indicated with half-arrows in (B). kbp, kilo–base pairs. (**D**) Western blots showing the modification of the Gap2 protein (*Synpcc7942_1742*) upon tagging. Blots for the Rubisco large subunit (*Synpcc7942_1426*) are shown as a loading control. (**E**) Bar graph showing the efficiency at each step shown in (B) and the overall success rate of the pipeline.

To advance our understanding of the *S. elongatus* proteome, we developed an approach to scarlessly tag proteins at their native C termini in a way that would facilitate the determination of each protein’s subcellular localization, relative abundance, and interacting proteins (Fig. 1A). We used a modified Golden Gate cloning approach to construct plasmids (Fig. 1B), such that the constructs’ integration into the genome by homologous recombination results in the 3′ tagging of the gene with the bright monomeric green fluorescent protein (GFP) mNeonGreen (mNG) and a 3xFLAG epitope, as well as the introduction of positive and negative selectable markers downstream of the gene of interest and flanked by copies of mNG to enable subsequent marker removal via a second homologous recombination event (Fig. 1B).

We transformed *S. elongatus* with each plasmid individually and selected for transgene integration based on kanamycin resistance, conferred through the positive selection marker *nptI*. As *S. elongatus* is polyploid, we used increasing concentrations of kanamycin to ultimately isolate homoplasmic “marked” (i.e., fully segregated) mutants, in which every chromosomal copy of the gene of interest was modified. To remove both selection markers to create “markerless” tagged mutants, we induced the excision of the selectable markers via a second round of homologous recombination of the flanking mNG genes using 5-fluorocytosine (5-FC), which is hydrolyzed to cytotoxic 5-fluorouracil by the *codA* gene product (negative selection). We tested our pipeline on 400 target genes using 96-well plate formats for cloning, transformation, and selection steps (table S1). Target genes were selected on the basis of them being associated with photosynthesis [see (*19*) for a review], the PlastidCut (conserved proteins in plastid containing organisms) (*20*), the CO_2_-concentrating mechanism (CCM) (*21*), low CO_2_ up-regulated (*22*, *23*), essentiality (*24*), as subcellular markers, plus other processes of particular interest to the cyanobacteria research community. Homoplasmic integration of the tagging construct and successful marker removal were validated in each case by colony polymerase chain reaction (PCR) (Fig. 1C), and introduction of the mNG and FLAG tags caused an ~30-kDa increase in observed protein molecular weight (Fig. 1D).

Each step in the plasmid cloning and selection of marked mutants had a success rate of between 91 and 99%, allowing us to generate 346 successfully mNG integrated mutants and to isolate homoplasmic tagged mutants for 330 (82.5%) of our target genes (Fig. 1E and table S1). Sixteen of the 346 integrated mutants failed to fully segregate potentially due to the fusion protein disrupting an essential function or tagging construct integration disrupting downstream expression of an essential gene (Fig. 1E). Our tagging success rate is comparable to other large-scale endogenous tagging studies (*11*, *14*, *15*) and higher than the ~53% exogenous tagging success rate in the photosynthetic alga *C. reinhardtii* that faces considerable gene cloning challenges due to intron essentiality and high GC content (*16*, *17*).

### Protein localization in markerless mutants

**Fig. 2.**
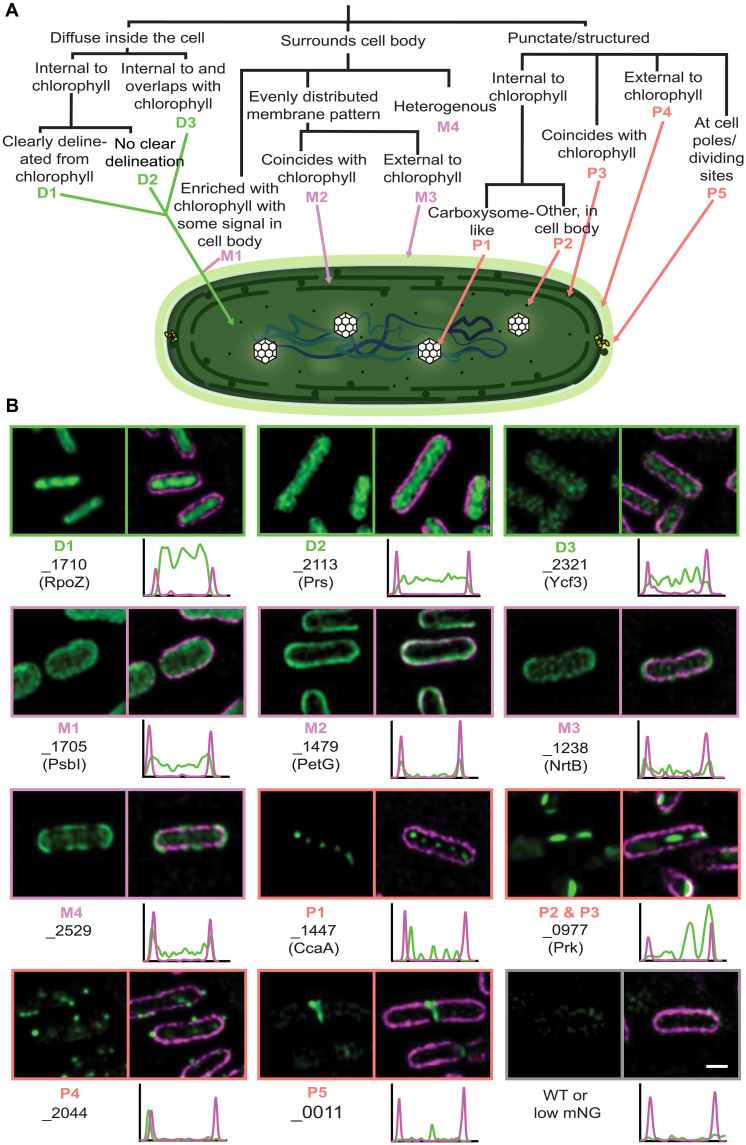
Subcellular locations of tagged proteins in *S. elongatus* PCC 7942. (**A**) Schematic overview of a decision tree for assignment of proteins to classes (or combinations of classes) according to the localization of mNG fluorescence. (**B**) Representative examples of tagged proteins (green) localizing to each cluster in the set of *S. elongatus* markerless mutants. Chlorophyll autofluorescence signals (magenta) are overlaid in the right-hand panels. Underneath each overlay the profile of fluorescence intensity across the length of the displayed cell is represented. The transects and raw values from these analyses are displayed in fig. S1. Scale bar, 1 μm.

**Fig. 3.**
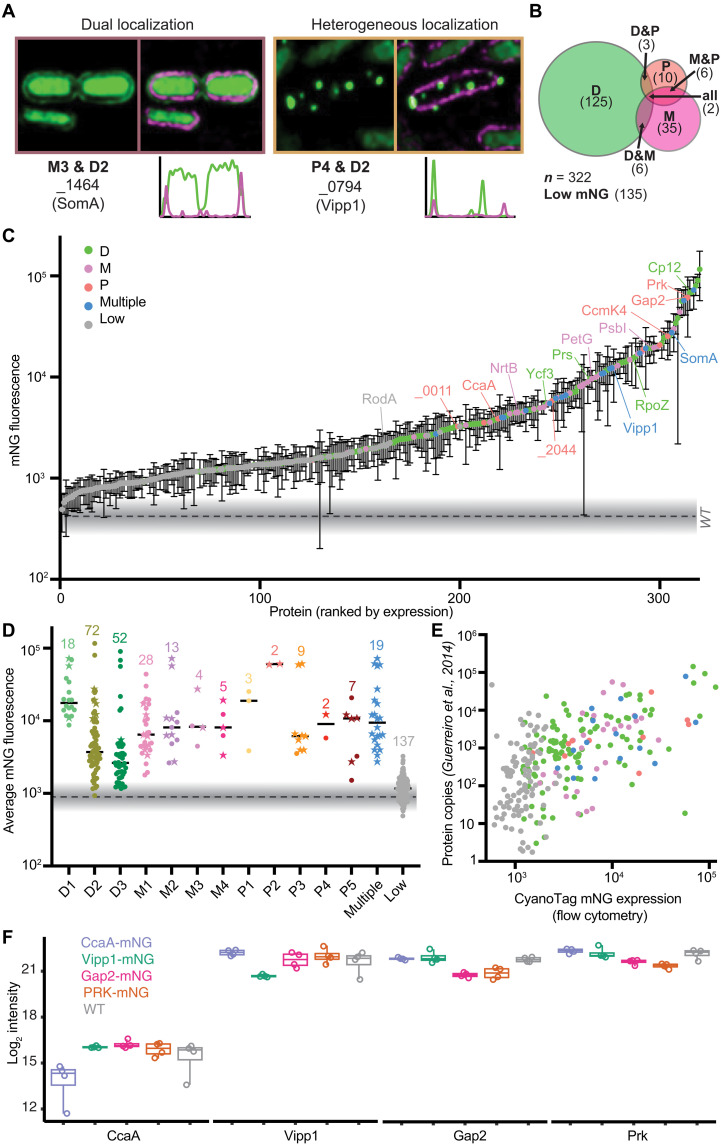
CyanoTag lines facilitate interrogation of protein expression and localization in live cells. (**A**) Example images of lines with multiple expression locations (see Fig. 2), either within the same cell (left) or with different patterns in different cells. (**B**) Venn diagram summarizing broad localization descriptors, and the crossover between them, for the imaged lines. (**C**) Normalized fluorescence intensities for markerless lines. Means and SDs for 3 or more replicates are plotted. (**D**) Mean fluorescence values of markerless lines, by localization category. Star-shaped points indicate that multiple localization descriptors are used. (**E**) Scatterplot of estimated protein copy number per cell (averaged across a 48-hour time course of 12-hour/12-hour light/dark cycles from a previously published study) plotted against the mean fluorescence intensity recorded in the corresponding markerless CyanoTag line. (**F**) Protein abundance determined by mass spectrometry of four mNG-tagged lines and wild type (WT). The upper and lower boundaries are the first/third quartile, respectively, and whiskers extend to minimum and maximum values. See table S2 for statistical comparisons.

We used super-resolution structured illumination microscopy (SIM) to observe mNG fluorescence in 322 markerless mutants. To aid classification of the fluorescent protein distribution for each tagged cyanobacterial strain, we built a decision tree based on the pattern of mNG fluorescence and how it relates to the consistent pattern of cellular autofluorescence, which primarily arises from the pigments in the thylakoid membranes (Fig. 2A). We used this to describe the patterns of fluorescence observed in each line using one or a combination of descriptors relating to diffuse patterns (D), membrane patterns (M), or puncta (P) (Figs. 2B and 3, A and B, fig. S1, and table S1).

Among these images, we observe a range of distinctive and/or previously undescribed localization patterns. For instance, using three lines where known carboxysome proteins were tagged, we see a characteristic punctate pattern of mNG fluorescence along the central axis of the cell (P1) (Fig. 2B). In multiple other examples, we show previously undescribed localizations for poorly characterized cyanobacterial proteins such as Synpcc7942_2044 (P4) (Fig. 2B). Little is known about Synpcc7942_2044, beyond its likely essentiality (*24*) and as containing a PRC-barrel (photosynthetic reaction center–barrel), which are speculated to have been acquired from purple bacteria and incorporated into the regulation of cyanobacterial electron transport (*25*). Its localization to distinct puncta external to the thylakoids hints either to a previously unknown regulatory mechanism or a different function entirely.

A small proportion (<9%) of the proteins we describe here do not fit neatly into a single localization category (Fig. 3, A and B, and fig. S1). The Vipp1 protein (Synpcc7942_0794) is one such example, where, under our imaging conditions (low-light), we see a disperse cytoplasmic localization of tagged Vipp1 protein in most cells but very bright puncta at the cell periphery in others (Fig. 3A). On the basis of previous observations, it is likely that, in these cells, Vipp1 is playing a role in maintaining the integrity of the thylakoid membrane and/or protecting the cell from stress-induced damage (*26*, *27*).

Although most of our observed localizations match expected locations for known proteins (table S1), we do see exceptions. Two notable ones are carboxysome shell components CcmP and CcmL. In our study, we see that fully segregated CcmP-mNG and CcmL-mNG show diffuse patterning instead of clearly defined puncta as expected for carboxysomes (see CcaA in Fig. 2B). This aligns with a previous study where yellow fluorescent protein (YFP) natively tagged and fully segregated CcmP and CcmL also failed to give clearly defined carboxysome patterning (*28*). However, partial segregation of CcmP-YFP (*28*) or CcmP-cerulean expressed from a neutral site (*29*) led to a clear carboxysome pattern. As carboxysome shell proteins are tightly packed to form a lattice, this indicates that steric hindrance of the tag may affect correct shell assembly, particularly when all copies are tagged.

### The CyanoTag pipeline enables screening of in vivo protein abundances

Because of the maintenance of both native cis- and trans-regulatory elements in the markerless lines, we hypothesized that mNG-tagged proteins would be at a similar abundance to nontagged versions, enabling mNG to act as a strong proxy for protein abundance. On the basis of this property, we developed a simple flow cytometry–based fluorescence assay (fig. S2) and used it to determine the relative abundance of proteins in live cells under standard growth conditions (Fig. 3, C and D). Fluorescence intensity was a strong predictor that protein subcellular location could be assigned from imaging data (Fig. 3, C and D). The trends in expression levels we observe in our flow cytometry data broadly reflect those observed in a published mass spectrometry datasets (Fig. 3E) (*30*). To further explore whether protein tagging had an impact on protein abundance, we performed label-free data-independent acquisition (DIA) proteomics (*31*) on four tagged lines plus wild type in quadruplet (table S2). Unexpectedly, we saw a systematic reduction in protein level of ~50% (34 to 58%) in the tagged lines (Fig. 3F and fig. S3). In two of the tested tagged lines, Prk and Gap2, that form a known complex [(*32*) and see below] we saw a coreduction of the complex partner. Thus, indicating that scarless protein tagging by integration of a fluorophore at the native genomic position can affect the absolute protein level. Whether this is specific to certain proteins or a more global effect requires further exploration. The impact of tagging has been assessed at a global level in yeast where it has been shown to lead to a significant decrease in abundance for ~16% of the proteome (*33*). Further exploration of the data revealed two consistently up-regulated proteins in the mNG-tagged lines (fig. S3, B and C). Synpcc7942_2576 is over 60-fold up-regulated in mNG-tagged lines and is an uncharacterized protein that contains a peptidase M50 domain (Pfam: PF02163) and Synpcc7942_2071 is an ATPase (adenosine triphosphatase) homolog of the type II/type IV secretion system that plays a critical role in protein secretion and is essential for pili formation (*34*). Although speculative, this suggests the presence of a mechanism to degrade and secrete fluorophore-tagged proteins in *S. elongatus* potentially due to changes in protein stability.

In conclusion, our flow cytometry–based approach gives a robust indication of relative protein abundance, providing a platform by which changes in protein expression, and cellular morphology, can be assessed in a high-throughput manner (examples in later sections). However, caution needs to be taken when interpreting absolute protein levels as fluorophore tagging can affect protein abundance.

### The CyanoTag pipeline facilitates comprehensive mapping of protein interactions

**Fig. 4.**
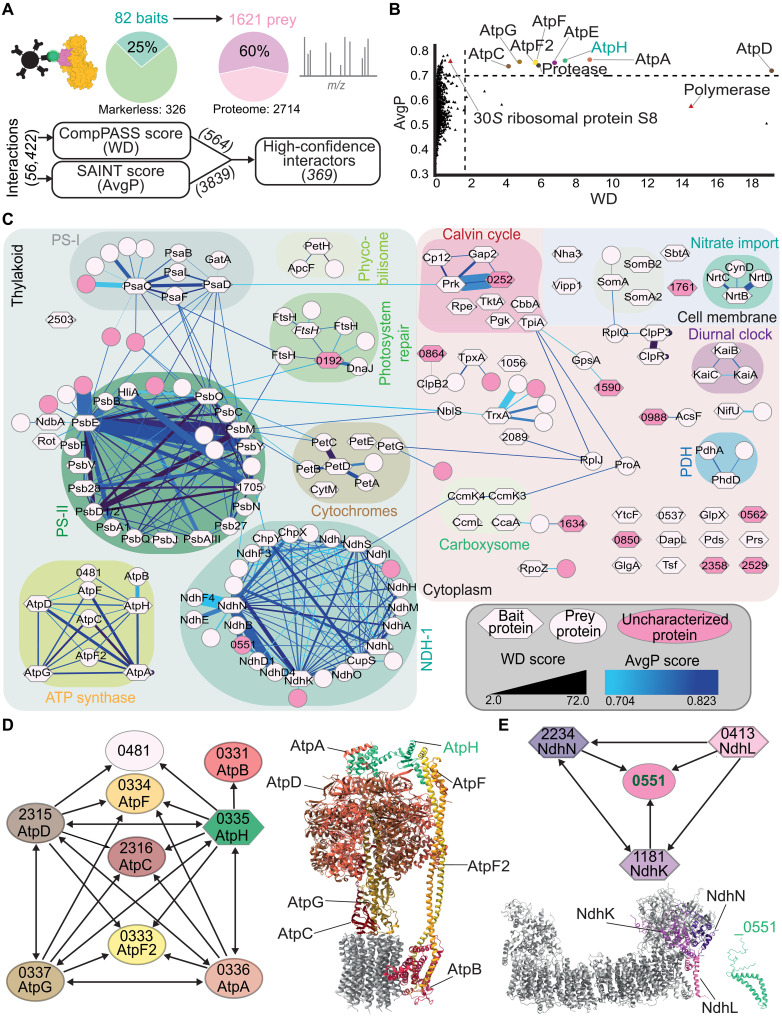
Preliminary *S. elongatus* protein interactome using CyanoTag lines. (**A**) Overview of the AP-MS and bioinformatic filtering pipeline, which was used to identify high-confidence protein interactions from a biologically diverse range of bait proteins. (**B**) Representation of bioinformatic filtering of protein interactions for the bait AtpH, a well-characterized component of the ATP synthase complex. Using just one confidence score (WD or AvgP) results in the presence of likely false-positive interactions (red triangles). Combining both preserves almost all previously characterized complex interactions (labeled). (**C**) Preliminary protein interactome of *S. elongatus* based on data from 82 bait proteins run in duplicate. Labels on nodes denote proteins by an abbreviated name if one exists or by their four number gene identifier otherwise. Node shape corresponds to bait or prey status, and edge width and color represent WD and AvgP scores, respectively. Known complexes are highlighted by the background color, and uncharacterized protein nodes are shaded pink. (**D**) Interactions of the bait protein AtpH (highlighted green) with its high-confidence preys (left). Model of the ATP synthase complex [Protein Data Bank (PDB): 6OQR] highlighting bait and prey proteins (right). (**E**) Interactions of the prey protein Synpcc7942_0551 (green) and the three bait proteins that it copurified with as HCIs (NdhN, L and K) (top). AlphaFold2 predicted structure of Synpcc7942_0551 and model structure of the NDH1-MS complex (PDB: 6TJV) with interacting subunits highlighted (bottom).

To explore the protein-protein interaction network in *S. elongatus*, we developed an affinity purification–mass spectrometry (AP-MS) method and applied it in duplicate to an initial cohort of markerless CyanoTag lines (Fig. 4A and table S3). We then developed a bioinformatic pipeline to filter mass spectrometry data, using a combination of CompPASS (*35*) and SAINT (*36*) analyses to define high-confidence interactors (HCIs) for each of the 82 bait proteins (Fig. 4, A and B). The data collated from these initial bait proteins resulted in the identification of two or more peptides corresponding to 1617 unique prey proteins representing ~60% the entire *S. elongatus* proteome (Fig. 4A). Of the 56,422 protein interactions detected, after filtering, 369 passed the thresholds to be HCIs. These data were compiled into an initial protein interactome comprising 82 baits and 135 unique prey proteins, which includes previously characterized interactions and highlights previously unidentified ones (Fig. 4C and fig. S4).

The resulting interactome map includes multiple interactions within and between known large protein complexes, including photosystems I and II (PS-I and PS-II, respectively), the NDH1 complex, nitrate import proteins, and the circadian clock regulatory proteins KaiABC (Fig. 4C). In cyanobacteria, the NDH1 complex is found in four isoforms, all that share a core common module but have different isoform-specific subunits (*37*). Using the core structural component NdhN (Synpcc7942_2234) as bait, 18 other core or isoform-specific NDH1 subunits were identified as HCIs (Fig. 4C). Using the AtpH subunit (Synpcc7942_0335) of the adenosine 5′-triphosphate (ATP) synthase complex as a bait, we detected HCIs with almost all of the known proteins of this highly conserved complex (Fig. 4, B to D) (*38*). Only subunits of the membrane integral C-ring were not classified as HCIs of AtpH. All four subunits of ATP synthase used as baits each identify interactions, almost exclusively, with multiple other subunits of the complex (Fig. 4, C and D).

Among the HCIs are several examples of previously undescribed interactions. Synpcc7942_0551 is an uncharacterized prey protein identified as interacting with three NDH1-MS complex subunits (Fig. 4, C and E). The interacting NDH1 subunits are adjacent to each other and in close proximity to the plastoquinone-binding site of the complex (Fig. 4E). This suggests that the AP-MS pipeline could be sufficiently powerful to resolve previously undescribed interactions to specific regions within a protein complex. Synpcc7942_0551 appears to be conserved across many cyanobacteria species and is structurally consistent with being a transmembrane protein, however is absent in *Thermosynechococcus elongatus* BP-1, the thermophilic cyanobacterium used for structural determination of the of the NDH1 complex (*39*, *40*). Our interactome strongly complements available large-scale protein complex maps generated by cellular fractionation followed by MS approaches in *Synechocystis* sp. PCC 6803 (*41*, *42*).

### Analysis of CyanoTag lines provides insights into the regulation of the Calvin cycle

**Fig. 5.**
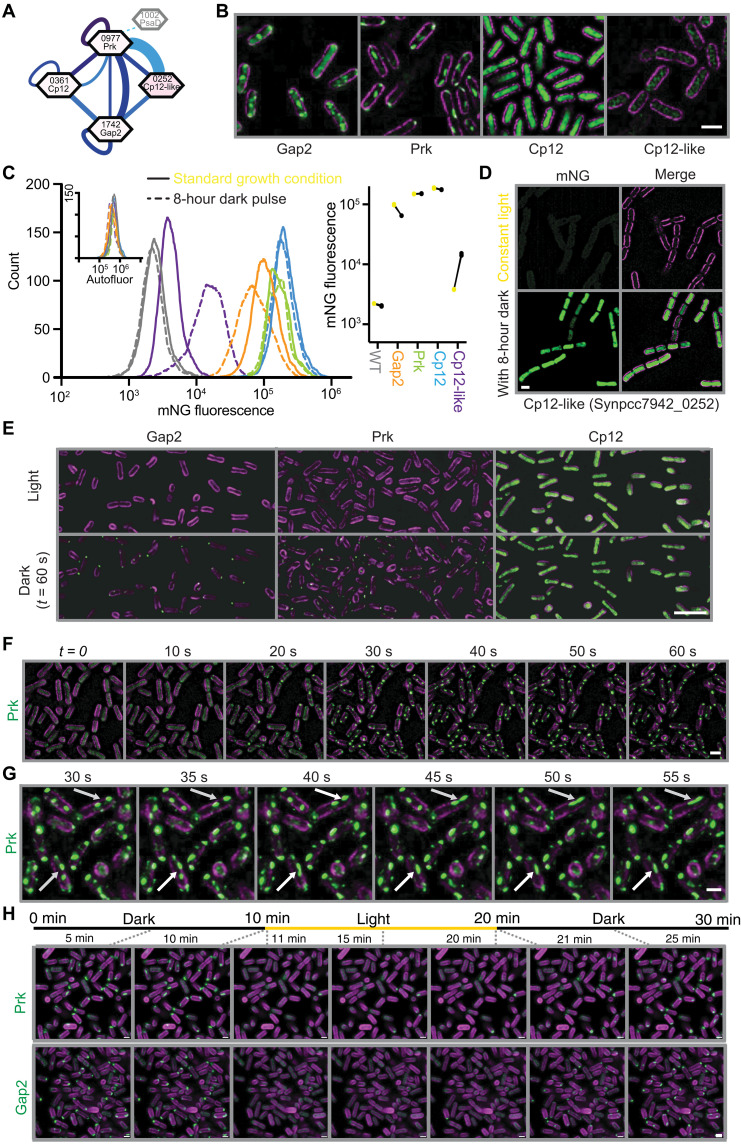
CyanoTag mutants allow visualization and investigation of light-dependent regulation of the Calvin cycle. (**A**) Interactions detected between Gap2, Prk, Cp12, and Cp12-like (Synpcc7942_0252) by our AP-MS pipeline. (**B**) Images of Gap2, Prk, Cp12, and Cp12-like collected under standard conditions (grown in constant light and then exposed to at least 5 min of darkness in the imaging chamber). (**C**) Flow cytometry–based quantitation of the mNG fluorescence of these proteins with and without 8-hour incubation in the dark. Left: Histograms of mNG fluorescence detected in a representative sample of each. Inset is the corresponding histograms showing the same samples’ autofluorescence (allophycocyanin channel). Right: Median mNG fluorescence of three replicates with light-incubated samples indicated by yellow points and dark-incubated in black. (**D**) Images of markerless Synpcc7942_0252-mNG–tagged populations with and without 8-hour incubation in the dark. (**E**) Images of populations of Gap2-, Prk-, and Cp12-mNG–tagged *S. elongatus* markerless lines before and after 1 min in the dark. (**F**) Frames of time-lapse microscopy showing rapid relocalization of mNG-tagged Prk protein upon the removal of light. (**G**) Frames from time-lapse microscopy of the fusion of Prk-mNG-containing puncta. Arrows indicate puncta that coalesce within this time frame. (**H**) Dark-light-dark transition of Prk- and Gap2-mNG. Images were taken every 1 min to minimize laser exposure. Scale bars, 1 μm.

In the course of developing the CyanoTag pipeline, we observed many notable or unexpected protein localization patterns in our fluorescently tagged lines, as well as evidence for previously undescribed protein-protein interactions. Among these were observations related to the regulation of the Calvin cycle, where we identified Synpcc7942_0252 as a potential interactor of Gap2 and Prk (Figs. 4C and 5A). This protein contains a Cp12-like domain but appears from our imaging and expression data to be present at a level much lower than Cp12 (Fig. 5, B and C). Using our flow cytometry method, alongside fluorescence microscopy, we detected increased fluorescence of mNG-tagged Synpcc7942_0252 after several hours of darkness (Fig. 5, C and D), corresponding with published analysis that showed the transcription of the corresponding gene to be among the most highly up-regulated in response to a 2-hour dark pulse (*43*).

Cp12 is a known regulator of the Calvin cycle, thought to bind Gap2 and Prk to sequester them away from the cycle under conditions nonoptimal for sugar production (*32*). Tagging of Gap2 and Prk with mNG allowed us to visualize this process in real time, such that we could show the rapid relocalization of these tagged proteins from a diffuse localization pattern to distinct puncta within seconds of light being removed (Fig. 5, E and F, and movies S1 to S3), which was in agreement with a previous study using *Synechocystis* sp. PCC 6803 (*44*). In *S. elongatus*, puncta are highly dynamic and able to undergo fusion (Fig. 5G and movie S4) and are rapidly reversible for both Prk and Gap2 (Fig. 5H and movies S5 and S6). These dynamic properties are widely associated with biomolecular condensates that form through liquid-liquid phase separation (*45*); however, further in vivo and in vitro supporting data are required to ascertain this (*46*). Puncta still form in the absence of Synpcc7942_0252 (figs. S5, A and B) that we observed to interact with Gap2 and Prk, indicating that this interaction is not essential to the dark-induced protein relocalization. This is in agreement with the low expression of Synpcc7942_0252 during the rapid relocalization of Gap2 and Prk observed upon light to dark transition and would suggest that Synpcc7942_0252 has a role after Gap2/Prk puncta formation (Fig. 5C). Cp12-mNG dynamics were less clear, potentially due to the high diffuse fluorescence level throughout the cytosol in the light masking the relocalization of a fraction of the Cp12-mNG population (Fig. 5E).

### Protein tagging provides further clues to protein function

**Fig. 6.**
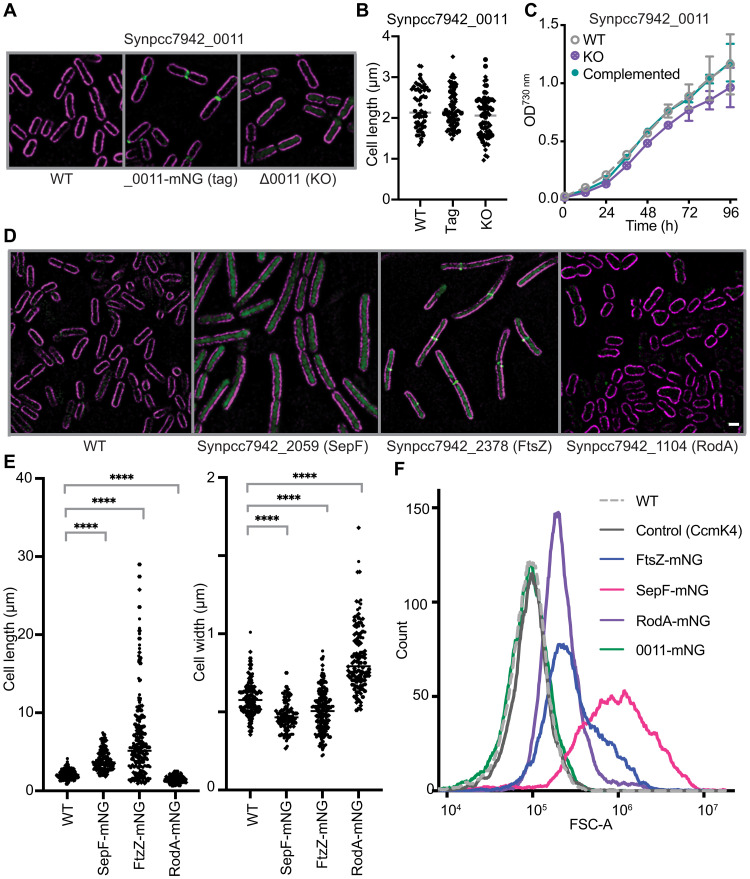
CyanoTag mutants provide insights into mediators of cell division and morphology. (**A**) Fluorescence microscopy showing *S. elongatus* populations with tagged (middle) and disrupted *Synpcc7942_0011* loci (right). (**B**) Analysis of cell dimensions of populations shown in (A). Data points represent one of at least 25 cells measured from each group in each of two experiments. (**C**) Growth of wild type, *Synpcc7942_0011* knockout (KO), and complemented lines measured by absorbance of populations at 730 nm. h, hours. (**D**) Fluorescence microscopy of markerless CyanoTag mutant targeting the *Synpcc7942_2059*, *Synpcc7942_2378*, and *Synpcc7942_1104* loci. Where Synpcc7942_2378 (FtsZ) is tagged, sites of mNG signal density at potential cleavage furrows likely correspond to the z-ring structure. (**E**) Analysis of cell dimensions of populations in (D). At least 100 cells measured per condition across three experiments. ****P < 0.0001, one-way analysis of variance (ANOVA). (**F**) Forward scatter (area: FSC-A) parameter of indicated cell populations measured by flow cytometry. Scale bar, 1 μm.

By tagging previously uncharacterized proteins, we stand to generate insights into where, and subsequently how, they function. For example, by tagging Synpcc7942_0011, we identified the brightest signals at the junctions between daughter cells as they separate from one another during the division process (Fig. 6A). This may indicate that Synpcc7942_0011 functions during the process of cell division. To investigate the function of Synpcc7942_0011, we used a modified CyanoTag plasmid vector to disrupt its genetic locus and create a knockout. Following the disruption of the *Synpcc7942_0011* locus (fig. S5A), we did not observe changes in cell size or shape by fluorescence microscopy (Fig. 6, A and B). Knockout lines were only slightly impaired in their growth rate (compared to wild-type and complemented strains), indicating that Synpcc7942_0011 is not required under our standard conditions (Fig. 6C). Further investigation is required to determine what role this previously uncharacterized protein is playing at sites of cell cleavage.

In addition to localization information, we observed several notable cell shape phenotypes during fluorescence imaging of three CyanoTag lines; tagging of Synpcc7942_2059 (SepF) or Synpcc7942_2378 (FtsZ) resulted in elongated cells, whereas tagging Synpcc7942_1104 (RodA) resulted in shorter, wider cells (Fig. 6, D to F). In bacterial cell division, the FtsZ protein forms a contractile ring at the midpoint of a growing cell, constricting to cause the separation of daughter cells. The SepF protein is thought to play a role in assembling FtsZ filaments and recruiting them to the cell membrane (*47*). Genetic disruption of these two proteins has been shown previously to result in elongated cells similar to those we observe in the corresponding CyanoTag lines (*48*). Synpcc7942_1104 is annotated as a functional homolog of RodA, a protein thought to be involved in cell wall synthesis and important for maintaining elongated cell shapes (*49*). The morphological changes we observe in these would be consistent, therefore, with the proteins’ functions being disrupted by the addition of the C-terminal mNG tag. Fusion tags’ influence on protein function is a widely recognized caveat of fluorescent protein tagging (*11*); however, as demonstrated here, this impairment does not always preclude the utility of these tagged lines and may, in contrast, provide further insights into protein function.

## DISCUSSION

### Insights and publicly available data and resources

We have developed the robust, high-throughput CyanoTag pipeline that can be used to generate *S. elongatus* cell lines expressing mNG-tagged proteins from their native loci. We demonstrated the use of these tagged lines to gain insight into a range of attributes for over 330 proteins, including their localizations, abundance, and interactions. Among the insights we have gained from studying our CyanoTag lines were real-time observations of spatial and temporal regulation of two key Calvin cycle proteins, Prk and Gap2. Our AP-MS studies also identified a potential previously undocumented interactor of Prk and Gap2, although whether/how it regulates Calvin cycle dynamics will require further investigation. In addition, the generated protein-protein interactome has robustly assigned proteins of unknown function to central, well-studied multimeric complexes, such as PS-II and NDH1. We were also able to identify a possible role in cell wall dynamics for a previously uncharacterized protein Synpcc7942_0011, initially clued via its notable localization at the division sites of separating cells.

In this work, we report our initial pilot study targeting 400 of the 2714 proteins encoded by the *S. elongatus* genome. With optimized high-throughput protocols, using a multiplexed format that has allowed considerable reductions in resource and plastics usage, this pipeline is readily scalable. We are already expanding our libraries, working toward greater coverage of the proteome, offering more opportunities to observe novel biology and generate the first experimentally defined whole cell interactome of a photosynthetic organism. Our data are available publicly via our online platform at https://morf-db.org/projects/York-Mackinder-Lab/MORF000032, which we will update as the project progresses and has the capability of integrating parallel datasets from compatible studies on *S. elongatus*. To further support the cyanobacterial research community, we are open to sharing our CyanoTag lines with other researchers and have also created and shared detailed protocols for each step in our pipeline (included in the Supplementary Materials).

### Limitations of our datasets

As with all high-throughput strategies, there are some important limitations to be considered when working with our datasets and tagged lines. Scarless tagging of proteins at their endogenous loci has the great advantage of keeping native regulatory elements intact but may still affect expression of downstream genes due to disrupting overlapping promoter sequences and may have hard to predict trans-regulatory effects on other genes. The addition of a 30-kDa tag at the C terminus of a protein has the potential to impair normal protein function, localization, interactions, and abundance. We see examples of these impacts in our dataset. In some cases, tagging precluded homoplasmic modification most likely through perturbing an essential function (e.g., with RbcS; table S1), led to a growth impairment (observed with SepF; Fig. 6), resulted in incorrect localization (e.g., CcmP and CcmL; table S1), or reduced protein abundance (Fig. 3F). However, this did not prevent us from tagging at least one copy of our target gene in over 90% of attempted transformations (Fig. 1E), observing correlation between our protein expression data and previously published estimates of protein copy number (Fig. 3E), confirming many previously determined localizations and assigning many new ones to previously unstudied proteins (table S1). Another known limitation of fluorescent protein tagging in bacteria is the correct translocation and subsequent folding of fluorophores that are translocated via the Sec pathway (*50*). In cyanobacteria, the Sec pathway translocates proteins in an unfolded state across both the thylakoid membrane and the cytoplasmic membrane (*51*), thus requiring correct folding outside of the cytoplasm. The oxidizing environment of the periplasmic space prevents efficient folding of GFP in *E. coli* (*50*), with this also appearing to be the case for three putative outer membrane proteins (SomA, SomA2, and SomB2; table S1) tagged in our study. Although SomA exhibited some signal external to chlorophyll suggesting a level of correct translocation and folding (Fig. 3A), SomA2 and SomB2 only showed cytoplasmic labeling, indicating poor translocation and/or a failure to correctly fold the mNG after translocation. Further additional limitations can be the cleavage of the tag after translation or transcription/translation initiation at an alternative site, with both potentially resulting in a separate population of mNG. Tag placement can also have a large impact on protein targeting and regulation. A systematic comparison of N-terminal versus C-terminal fluorescence protein tagging for ~1800 proteins in yeast showed that ~20% of proteins had a different localization depending on what terminus was tagged (*52*). In our study, we only performed C-terminal tagging and acknowledge that N-terminal tagging could influence assigned localizations for a subset of proteins. In several cases, tagging interfering with protein function allowed us to make inferences about the target protein’s role; for instance, an elongated cell shape implied a protein involved in cell separation had been disrupted (Fig. 6, E and F).

Where markerless mutants cannot be generated or are too greatly impaired, a possible alternative is to use nonhomoplasmic or marked CyanoTag lines maintained with kanamycin selection. In the course of developing the pipeline, we noted generally increased expression of mNG in marked mutants compared to their markerless counterparts (particularly when tagged protein expression was low; fig. S5D), suggesting that either some expression of the second mNG-3xFLAG module (Fig. 1B) can occur or that the efficient *rrnB* terminator (*53*) inserted downstream of the first mNG-3xFLAG consistently enhances gene expression/protein translation over the native terminator. Although this does not preclude the use of nonhomoplasmic or marked mutants in further studies, it is an important caveat of their use and is the reason our analyses in this manuscript focus on markerless lines.

The impact of the C-terminal tag combined with the conditions in which we have imaged CyanoTag lines also play into the confidence that can be placed in the localizations we have ascribed. The images in our library were collected using the same set of growth and imaging conditions, and as such, we are not necessarily observing protein localization under the conditions where each protein is optimally expressed or at the location where it functions. The nutrient composition of the growth media affects a range of proteins; for instance, the balance of plastocyanin and other cytochromes shifts based on copper availability (*54*); hence, our use of <300 nM copper in our media may explain why we see low expression of plastocyanin (table S1). Similarly, because we grew our lines in constant light, we observed low expression of the dark-induced Synpcc7942_0252 under our standard imaging conditions (Fig. 5D). Our observations of Gap2 and Prk also highlight how dynamic protein localization can be; these proteins play their primary role in the Calvin cycle in the cytoplasm but quickly relocalize to an alternate punctate location under our imaging conditions (Fig. 5, E, F, and H, and movies S1 to S6). We also acknowledge that, even with experimental replication, most of our images are a snapshot of one particular point during the log phase of each cell line’s growth and that our datasets are not capturing oscillations of protein expression that are known to occur even under constant growth conditions (*55*). We encourage the use of our tagged lines in further studies of specific proteins or responses to conditions.

### Perspective

Photosynthetic organisms play a central role in ecosystem stability and global biogeochemical cycling. There is a growing need to accelerate our understanding of photosynthetic microbes and to use this knowledge to address huge global challenges relating to food security, threatened ecosystems, and the escalating climate crisis. Through providing a holistic view of cellular protein localization, abundance, and interactions in response to changing external conditions, the CyanoTag project aims to help unlock the potential that cyanobacterial biology can offer to these vital areas.

## MATERIALS AND METHODS

### Cyanobacterial culture conditions

Mutants were generated on a wild-type *S. elongatus* PCC 7942 background. Cultures were maintained at 30°C under constant light conditions (50 μmol photons m^−2^ s^−1^) on 1.5% agar BG-11 media plates. Liquid cultures were maintained in 1-ml volumes of BG-11 in deep 96-well plates and shaken on an orbital plate shaker at 150 rpm.

### Plasmid design and Golden Gate cloning

Two CyanoTag template plasmids, pLM433 and pLM434, were constructed. Both plasmids contained two copies of the cytotoxin-encoding gene (*ccdB*) each flanked by *BspQ*I (pLM433) or *BsaI* (pLM434) restriction sites. Present between the *ccdB* sequences were a mNG/3xFLAG tagging module, a kanamycin selection marker (*nptI*), and a negative selection marker (*codA*) followed by a second copy of the mNG/3xFLAG module. At the 5′ end of the first mNG/3xFLAG module are 27 base pairs encoding a GDLGGSGGR flexible linker. To generate plasmids capable of integration at the 3′ end of each target gene, a Golden Gate cloning reaction was used to replace the *ccdB* sequences with ~800–base pair homology arms that had been amplified from genomic DNA by PCR.

### Generation and selection of mutants

*S. elongatus* PCC 7942 cultures were transformed by incubation with plasmids overnight at 30°C in the dark. Selection on BG-11 agar plates containing kanamycin (25 μg/ml) was used to isolate transformed cells, which were then grown in wells of deep 96-well plates in 1 ml of BG-11 medium containing kanamycin (25 μg/ml). Cyanobacteria were selected on increasing concentrations of kanamycin (50, 100, and lastly 200 μg/ml), and homoplasmic marked mutants were identified using colony PCR. Markerless mutants were then generated by homologous recombination between the two mNG/3xFLAG modules via selection on BG-11 agar containing 5-FC (0.1 g/liter). Homoplasmic markerless mutants were identified using colony PCR. *Synpcc7942_0011* and *Synpcc7942_0252* knockout lines were generated using similar methods; modified plasmids carrying a kanamycin resistance cassette flanked by homology arms corresponding to the target gene were transformed to induce insertional mutants (fig. S5A). To complement the *Synpcc7942_0011* knockout, the Synpcc7942_0011 coding sequence was amplified from the *S. elongatus* PCC 7942 genome and inserted into the pSyn_6 vector, which contains NS1 (neutral site 1) homologous recombination regions and a strong constitutive *psbA* gene promoter. Following transformation, colonies were selected with spectinomycin (50 μg/ml). Knockout and complemented lines were validated by colony PCR and sequencing.

### Fluorescence microscopy

Super-resolution imaging was performed using a Zeiss Elyra 7 microscope, using a lattice SIM^2^ method, with the “scale to raw image” parameter enabled. Fluorescent images were collected using a 63x/1.4 oil immersion objective lens. Both mNG and chlorophyll were excited using a 488-nm laser line, and their emissions split between two cameras for simultaneous capture. The mNG emission was captured at 495 to 550 nm, and chlorophyll emission was captured greater than 655 nm. To obtain whole cell information, *z*-stacks were collected with a step interval of 0.11 μm; each optical plane is composed of 13 phase images. The raw phase images were processed in the Zeiss Zen Black software using the SIM^2^ software module. The SIM^2^ module was set to process the images in three dimensions with the output set to scale to original input intensity. Misalignment between fluorescent channels were corrected by collecting a reference dataset of TetraSpeck microspheres (0.2 μm; Invitrogen, T7280), and a correction “Affine” matrix was calculated using the Zen Black “Channel Alignment” tool. This matrix was incorporated into the SIM^2^ module to correct for any misalignment. Images were further processed using Fiji (Image J). Comparison of the mNG and cellular autofluorescence (mostly from chlorophyll), signal distribution was used to determine subcategories of protein localization. Where the mNG signal was low and the resultant mNG localizations looked similar to that of wild-type cells, no localization descriptors were assigned. Fluorescence intensity profiles and quantifications of cell dimensions were performed using the measurement tools in Fiji. For time-lapse imaging, samples were incubated in the microscope chamber under LED (light-emitting diode) light (50 μmol photons m^−2^ s^−1^) until immediately prior to recording. Time-lapse imaging of Gap2 and Prk localization during dark to light to dark transitions (Fig. 5H and movies S5 and S6) was performed on a Zeiss LSM980 MP Airyscan 2 microscope using a 63x/1.4 oil immersion objective lens. mNG was excited at 488 nm, and its emission was collected at 495 to 550 nm. Chlorophyll was excited at 693 nm, and the emission was collected at 655 to 735 nm. Airyscan Images were sequentially collected at 60-s intervals over a 30-min period. The samples were incubated in the blacked-out microscope incubation chamber for the first 10 min. To generate light, an external desk lamp was positioned inside the incubator and was manually switched on to illuminate the sample area (20 μmol photons m^−2^ s^−1^). After 10 min, the light was manually switched off. The Airyscan raw images were processed using Zen Blue, and these processed images were compiled using Fiji.

### Quantification of protein abundance

*S. elongatus*–tagged mutant colonies were transferred to 96-well microplates containing BG-11 medium and incubated at 30°C under light (50 μmol photons m^−2^ s^−1^) for 2 days, after which they were analyzed using a Cytoflex S cytometer. Live cells were gated based on their autofluorescence, and the median fluorescence intensity of this live cell population in the FITC (fluorescein isothiocyanate) channel was used as a proxy for mNG fluorescence.

### Affinity purification–mass spectrometry

Lines were prioritized for AP-MS based on having detectable protein expression, interesting localizations, and/or being involved in diverse subcellular processes. Tagged lines were grown in duplicate to log phase before being pelleted (~30 mg of pellets) and snap frozen in liquid nitrogen. Pellets were resuspended in an immunoprecipitation (IP) buffer [200 mM d-sorbitol, 50 mM Hepes, 50 mM KOAc, 2 mM Mg(OAc)_2_, and 1 mM CaCl_2_] containing protease inhibitors (Roche cOmplete EDTA-free), 2% digitonin, 1 mM phenylmethylsulfonyl fluoride (PMSF), 0.5 mM NaF, and 0.15 mM Na_3_VO_4_. Cells were lysed by agitation with 0.4- to 0.6-mm glass beads for 6 s followed by a 10-s incubation on ice for a total of 15 min. Lysate was incubated with anti-mNG nanobody–Trap Agarose beads (ChromoTek), according to the manufacturer’s instructions, for 60 min on a rotating platform at 20 rpm. Beads were washed three times with the IP buffer containing 0.1% digitonin followed by a final wash in the absence of digitonin. All incubation steps were carried out at 4°C. Purified proteins were digested on-bead with the addition of 100 ng of Promega sequencing grade trypsin (V5111) and incubation at 37°C overnight. Peptides were loaded onto EvoTip Pure tips for nano-ultra-performance liquid chromatography (nanoUPLC) using an EvoSep One system. A preset 100 SPD gradient was used with an 8-cm EvoSep C_18_ Performance column (4 to 8 cm by 150 μm by 1.5 μm). The nanoUPLC system was interfaced to a timsTOF HT mass spectrometer (Bruker) with a CaptiveSpray ionization source (Source). Positive parallel accumulation serial fragmentation DIA (PASEF-DIA), nano-electrospray ionization mass spectrometry (nanoESI-MS), and MS^2^ spectra were acquired using the Compass HyStar software (version 6.2, Thermo Fisher Scientific). Instrument source settings were as follows: capillary voltage, 1500 V; dry gas, 3 liters/min; and dry temperature, 180°C. Spectra were acquired between mass/charge ratio (*m/z*) 100 and 1700. DIA fragmentation of 25-Th windows were used covering *m/z* 400 to 1201 and an ion mobility range of 0.6 to 1.6 1/*k*_0_. Collision energy was interpolated from 20 eV at 0.5 V·s/cm^2^ to 59 eV at 1.6 V·s/cm^2^. Resulting data in .d format were searched using the DIA-NN software (version 1.8) against an in silico predicted spectral library generated from the *S. elongatus* PCC 7942 subset of UniProt. DIA-NN peptide identifications were compiled with KNIME and data filtered to 1% FDR (false discover rate). Two peptides (LATSPVLR and IAQVNLSR) likely corresponding to trypsin autolysis peptides were stripped from the results to prevent false positives.

### AP-MS data analysis

DIA-NN–derived, non-normalized protein group quantification values from all samples were run through a CompPASS package in R Studio and a continuous measurement variation of SAINT analysis in Ubuntu. Interactions that fell in both the top 1% WD score (CompPASS) and 7% AvgP score (SAINT) were defined as HCIs. These were inputted into Cytoscape for interactome generation.

### Whole cell proteomics

Wild-type and mutant lines were grown in quadruple to an OD_730_ (optical density at 730 nm) of 0.5 in Erlenmeyer flasks in BG-11 medium at 30°C under continuous illumination of 50 μmol photons m^−2^ s^−1^ on a shaker (150 rpm). Cells were harvested by centrifugation at 1500*g* at 4°C for 15 min. Thirty milligrams of pellets was flash frozen in liquid nitrogen and resuspended in the IP buffer containing 2% digitonin, protease inhibitors (Roche cOmplete EDTA-free), 1 mM PMSF, 0.5 mM NaF, and 0.15 mM Na_3_VO_4_. Subsequently, the cells were lysed by agitation with glass beads for 15 min in a 4°C room (repeating 6-s agitation followed by 10-s incubation on ice). Whole cell lysate samples as 20-μl aliquots were diluted 1:1 with aqueous 10% (v/v) SDS, 100 mM triethylammonium bicarbonate (TEAB). Protein was reduced with 5.7 mM tris(2-carboxyethyl)phosphine and heating to 55°C for 15 min before alkylation with 22.7 mM methyl methanethiosulfonate at room temperature for 10 min. Protein was acidified with 6.5 ml of aqueous 27.5% (v/v) phosphoric acid and then precipitated with dilution sevenfold into 100 mM TEAB and 90% (v/v) methanol. Precipitated protein was captured on S-trap (ProtiFi, C0-micro) and washed five times with 165 μl of 100 mM TEAB and 90% (v/v) methanol before digesting with the addition of 20 μl of Promega Trypsin/Lys-C mix (0.1 μg/μl; V5071) in aqueous 50 mM TEAB and incubation at 47°C on a hot plate for 2 hours. Peptides were recovered from S-trap by spinning at 4000*g* for 60 s. S-traps were washed with 40 μl of aqueous 0.2% (v/v) formic acid and 40 μl of 50% (v/v) acetonitrile:water and washes combined with the first peptide elution. Peptide solutions were dried in a vacuum concentrator and then resuspended in 20 ml of aqueous 0.1% (v/v) formic acid. PASEF-DIA liquid chromatography–mass spectrometry was performed as detailed for AP-MS samples with the exceptions that a 15-cm EvoSep C_18_ Performance column (15 cm by 150 μm by 1.5 μm) was used for separation over a 30 SDP preset EvoSep gradient. Protein identification and quantification were performed using DIA-NN (v1.9) as detailed for AP-MS samples, except that the high-accuracy setting for quantUMS was used in place of high precision.

## Supplementary Materials

### The PDF file includes:

Figs. S1 to S5 Legends for movies S1 to S6 Legends for tables S1 to S3 Supplementary Methods
